# AI Agents Are Coming: 5-Stage Taxonomy of Language-Based AI Systems for Psychiatry, Psychotherapy, and Counseling

**DOI:** 10.2196/91746

**Published:** 2026-07-13

**Authors:** Raphael Schuster, Constantin Yves Plessen, Per Carlbring, Andreas Walther

**Affiliations:** 1 Psychotherapy and Psychotherapy Research (CBT) Center for Psychotherapy University of Graz Graz, Styria Austria; 2 Department of Psychology Stockholm University Stockholm Sweden; 3 School of Psychology Korea University Seoul, Seoul Republic of Korea

**Keywords:** agentic AI, AI agent, AI ecosystem, artificial intelligence, blended therapy, chatbot, classification, framework, large language model, multiagent system, typology

## Abstract

The rapid evolution of large language models has accelerated the development of agentic artificial intelligence (AI) systems capable of pursuing autonomous goals, creating an urgent need for structural frameworks in psychiatry and psychotherapy. While existing classifications often draw parallels to autonomous driving, this paper argues that the mental health domain requires a distinct, domain-specific theoretical foundation, as the 2 domains differ fundamentally in their semantic, ideographic, and epistemological demands. Furthermore, they differ in their end goals, for which we introduce terms such as agentic guidance capability. To guide clinicians and researchers through these developments, we propose a 5-stage taxonomy for language-based AI systems that differentiates technical functionality from clinical effectiveness. The taxonomy progresses from level 1 (knowledge level), in which systems perform static benchmark tasks, to level 2 (elementary level), characterized by dynamic engagement in specific therapeutic microskills. At level 3 (integration level), systems achieve consistency across and within modules, as well as basic case-level conceptualization suitable for blended therapy under human oversight. Level 4 (saturation level) describes therapist-in-the-loop systems capable of autonomous functioning with minimal supervision, whereas level 5 (mastery level) represents AI systems that are technically capable of performing autonomous therapy. By distinguishing technical functionality from clinical effectiveness, we conclude that level 4 or level 5 performance does not automatically translate into full treatment effectiveness, even if high treatment fidelity can be achieved. We conclude by emphasizing the need to shift benchmarking from static knowledge tests to dynamic evaluations of therapeutic capabilities in order to safely navigate the transition toward autonomous care.

## Background

Artificial intelligence (AI) and machine learning constitute some of the fastest-growing and most promising technological paradigms of our time [1]. Although these technologies have long been transforming daily life “under the hood” (eg, through handwriting or speech recognition), their rapid development in recent years has brought them into the public spotlight. The emergence of large language models (LLMs), such as ChatGPT, represents a provisional peak: never before has a product been adopted so rapidly by so many people worldwide [2].

Given the relatively slow implementation of digital interventions, this development resembles a landslide—or even a black swan event [3]. We knew that AI would eventually emerge; visionaries such as Alan Turing [4] and John von Neumann speculated about this possibility even before the advent of modern computers. Stephen Hawking warned of its potential and far-reaching societal changes as early as the 1980s [5]. Yet the pace of recent developments has surprised even most experts and carries major implications for the near future. To illustrate these implications, we consider 2 contrasting examples: first, established health care systems, in which AI primarily raises questions of optimization, and second, care settings characterized by substantial underprovision.

The first example is the case of so-called just-in-time adaptive interventions (JITAIs) [6]. Considerable development effort and research funding have been invested in these systems, yet they now risk becoming overly technical in light of current developments. JITAIs must navigate a complex chain of proxies: psychological constructs must first be translated into numerical scales or other proxy measures, then extensively assessed and integrated through relatively simple, human-defined algorithms, ultimately yielding a limited set of behavioral recommendations. At each step, measurement error accumulates. The limited data streams and narrow mathematical state space used by these systems tend to produce rigid or unnatural applications. While advanced analytical methods, such as ML or Bayesian algorithms, could improve the extraction of relevant information [7], high data requirements, study heterogeneity, and contextual factors may, for example, limit the feasibility of large-scale data analysis or increase the risk of overfitting [8]. In contrast, LLMs, as powerful foundation models, possess an enormous mathematical state space, comprising billions of parameters [9], and can generate fluid dialogue as well as a wide range of dynamic intervention suggestions.

For this reason, LLM-based systems may be better suited to therapeutic contexts in which adaptation depends less on predefined numerical proxies and more on semantic understanding, contextual responsiveness, patient-specific relational and contextual knowledge, and fluid dialogue. For example, rather than relying solely on abstract activity targets, such a system could incorporate personally meaningful relational and routine-based information—such as knowing that Kiara is the patient’s partner, that they usually go running together on Wednesdays, and that Friday may be a particularly suitable time for an additional run because the patient finishes work earlier and is entering the weekend. It could therefore suggest asking Kiara whether she would like to go for a run. Such capabilities might be conceptualized as just-in-topic adaptive interventions or, more broadly, as forms of semantic adaptive intervention.

Ultimately, however, the clinical relevance of JITAIs still needs to be established more clearly [7]. If a positive treatment impact can be demonstrated, AI-supported hybrid strategies may represent a promising path forward. In this context, algorithms for treatment planning and personalization in routine care will likely also be integrated with AI [8].

The second example of the unprecedented speed, scalability, and current off-label reach of these systems can be found in war-torn Ukraine [10]. In a recent survey of 2000 individuals conducted by the Kyiv International Institute of Sociology [11], only 3.9% reported having consulted a psychologist or psychiatrist. A similar proportion (2.8%) stated that they had turned to LLMs for psychological support. Extrapolated to the population, this would amount to roughly 1.1 million people seeking help from LLMs. For comparison, the World Health Organization (WHO) states in its latest annual report [12] that it reached 4.7 million Ukrainians with various forms of medical support in collaboration with 114 partner organizations. Although these figures should not be compared directly, they nevertheless highlight the enormous diffusion of this technology into society and the efficiency of this form of off-label assistance.

## Past, Present, and Future of AI Agents, Agentic AI, and Multiagent Systems

While the idea of AI can be traced back to the 1950s—such as the 1955 research proposal on AI by McCarthy et al [13], and the 1958 discussion of machines as rational problem solvers in *Programs With Common Sense* by McCarthy [14]—the term AI agents first appeared in the 1990s in the work of Wooldridge and Jennings [15], as well as in the explicit formulation of the agent–environment paradigm by Sutton and Barto [16]. At that time, AI agents were typically conceptualized as single entities or systems exhibiting agentic behavior, although early concepts of cooperative distributed AI had already begun to emerge [17]. This development led to early conferences such as the International Conference on Multi-Agent Systems (ICMAS) in 1995, by which time authors such as Wooldridge and Jennings were already referring to multiagent systems as an established concept [15,18,19]. These systems generally relied on symbolic and rule-based approaches, which were clearly limited in their learning capacity and flexible feature extraction. At the same time, computationally constrained probabilistic, reinforcement-based, and neural approaches already existed [20,21]. These developments also gave rise to the question of how multiple agents could best be coordinated or orchestrated [22,23].

More recently, computationally powerful large neural networks, transformer architectures, and gradient descent–based learning methods [8], and probabilistic and generative models have undergone rapid development. In the context of this paper, generative LLMs usually form the core component of AI agents and agentic AI systems and are central to language processing, interpretation, and response generation.

**Figure 1:**
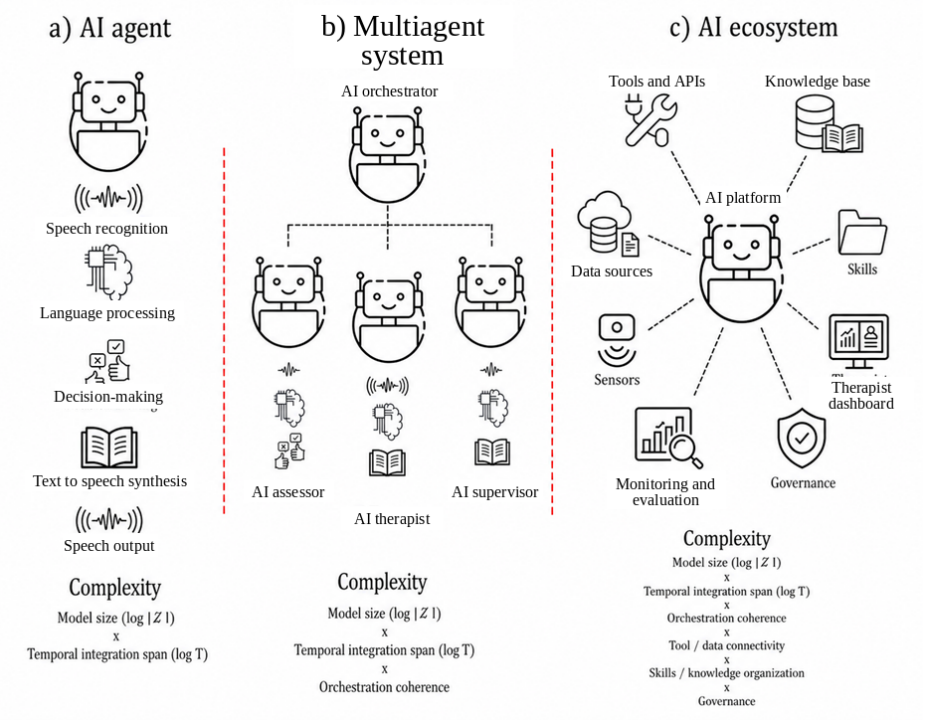
Schematic picture of components of artificial intelligence (AI) agents, agentic AI (multiagent framework), and AI ecosystems. AI agents as single entities can operate on certain tasks. Agentic AI allows integration of several complex tasks (eg, by multiagents or ecosystems). In language-based psychiatric contexts, this may include psychological conversation, supervision of dialogue, integration of therapy progress, or signals such as assessments or sensor data. The exact model components depend on model design (eg, architectural constraints or learning paradigms such as frequentist or Bayesian updating, or the type of timewise compression) and functionality. Schematic description of model complexity as a function of model components and temporal integration. Orchestration, tool/data connectivity, skills (eg, capabilities implemented as classical algorithms), and governance form part of model size and are only depicted separately for illustration. API: application programming interface.

While the term AI agent usually refers to a single entity operating with a specific functional scope (eg, a chatbot for dialogue-based interaction), multiagent systems operate through multiple agents and typically require at least 1 additional entity to orchestrate their interaction. Such systems are particularly useful when complex tasks (eg, an AI copilot for therapy) need to be decomposed into different subtasks (Figure 1).

The term agentic AI is used in the context of comparatively powerful, modern, and iteratively operating AI systems (autonomously pursuing goals through iterative cycles of reasoning, planning, tool use, and environmental interaction). It was popularized in AI discourse around 2024 by prominent experts such as Andrew Ng [24]. In psychological treatment, such systems may appear as a single entity to the patient, while in the background, one or several AI agents—or even multilayered AI ecosystems with a range of capabilities and levels of computational depth—carry out typical treatment-related tasks, such as psychological dialogue, symptom assessment and progress monitoring, the application and evaluation of therapeutic techniques and strategies, and the supervision and coordination of the overall process.

A key factor in the long-term development and possible acceleration of agentic AI may be the creation of self-improving training loops, for example, within in silico simulation environments [25]. Similar paradigms have already been explored in fields such as autonomous driving and robotics [26,27]. In relatively closed domains with clear feedback signals, including chess, Go, poker, and first-person video games, such approaches have enabled rapid and, in some cases, superhuman performance gains.

As will be explained in the following paragraphs, however, the psychological domain differs fundamentally from these settings. It is less formally specified, more context-sensitive, and considerably harder to evaluate using stable or objective metrics. Furthermore, as discussed in the section Domain-Specific Benchmarking vs the Autonomous Driving Metaphor, the ultimate aims of strong agentic AI in psychology may be more complex, and full autonomy may be less central—or may need to be conceptualized differently.

That said, early forms of automated patient–therapist simulation, self-play, and automated fine-tuning are beginning to be explored [28-31]. Challenges and limitations include restricted variability in responses and difficulties in dialogue evaluation [28], the rapid development of underlying language models [29], limited dialogue depth and temporal coherence across sessions [30], and unrealistic therapist behavior as well as limited reinforcement learning–based fine-tuning [31]. There are also systems that simulate only patients or only therapists and are proposed primarily for training purposes [32-34]. Overall, many of these studies remain at a conceptual stage (eg, conference papers or non–peer-reviewed literature), or they rely on LLMs rather than agentic AI, which likely reflects both the novelty of the field and the rapid pace of its development. Given the far-reaching implications and potential relevance for mental health care, this domain of research appears particularly important.

## Language-Based AI Agents for Mental Health

While many users are still discovering the utility of LLMs—and developers are contemplating their economic implications—progress is already moving toward AI agents. Agents are systems designed to formulate goals and subgoals and pursue them autonomously. In doing so, many of their core functions rely on well-established LLMs based on transformer architectures for data encoding and representation (compression). Other approaches implement active inference [35], which provides a framework for modeling a basic behavioral loop characteristic of living beings: actively seeking or perceiving information, analyzing and representing it, and acting on the basis of that representation, consistent with concepts of embodied intelligence and active inference. Building on such basic functionalities, even higher-order cognitive capacities—such as exploratory behavior or rudimentary forms of machine awareness [36]—may emerge.

Agentic behavior constitutes a critical milestone for several reasons. To date, agency has primarily been attributed to living beings. For artificial systems to possess agency, they must be capable of pursuing goals—potentially even self-generated ones. When agency coincides with high capability, the question of responsibility becomes correspondingly more pressing. High-stakes autonomous decision-making is no longer limited to science fiction; it is already a reality in domains such as autonomous weapon systems.

In psychiatric and psychological practice, reliable agents would initially imply that the therapeutic dyad becomes a kind of triad. Patients could have digital assistants available 24/7, capable of supporting therapeutic processes between sessions. Some hope that agents may ultimately realize the long-pursued goal of e–mental health: whereas traditional digital interventions without therapeutic guidance (eg, internet-based cognitive behavioral therapy [iCBT]) have at times produced poorer outcomes than face-to-face therapy [37], agents might eventually be able to support—or even autonomously manage—the entire therapeutic process. In this regard, however, it is important to acknowledge a potential shift in how the guided-unguided gap is assessed for certain mental disorders, as recent work suggests more nuanced differences between the 2 modalities, particularly with respect to long-term effects and applications outside Western countries [38].

The counterargument is that such systems remain therapeutic tools and do not generate the interpersonal commitment that arises uniquely between 2 human beings. Given the extensive evidence supporting self-help approaches and unguided eHealth interventions [39], it currently seems reasonable to assume that a proportion of engaged patients may achieve meaningful improvement. However, the overall group-level effects of guided or blended approaches involving a human therapist are likely to remain superior for the foreseeable future.

**Figure 2:**
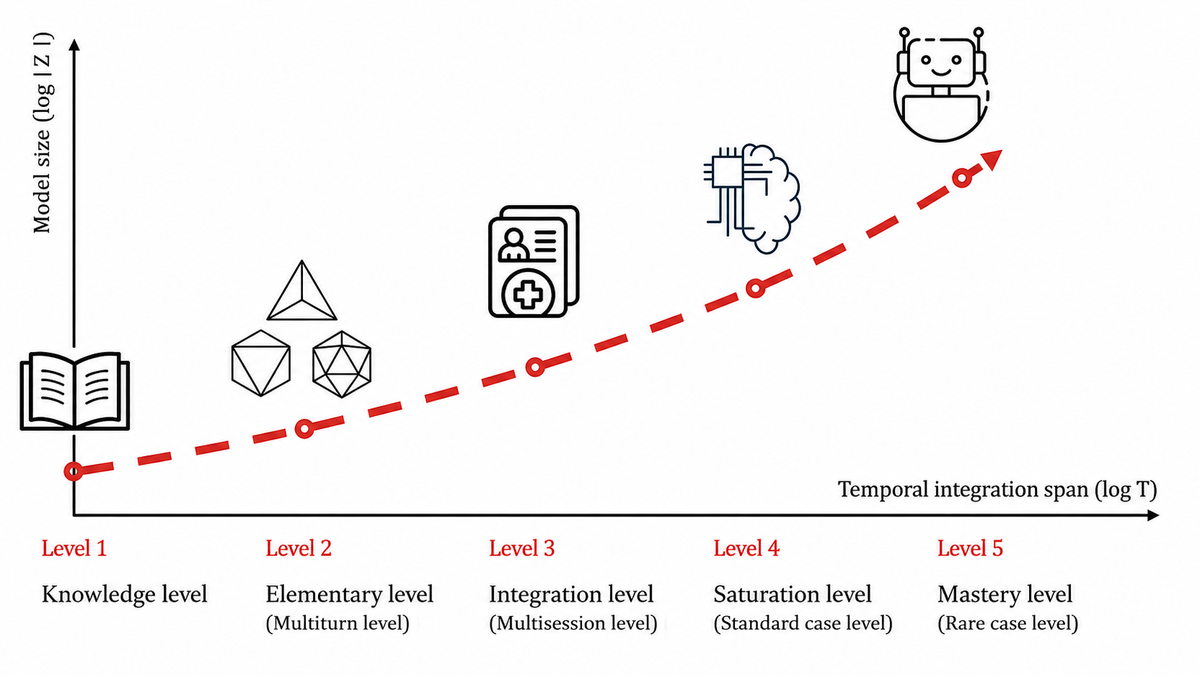
The 5 stages of language-based artificial intelligence (AI) systems for psychiatry, psychotherapy, and counseling. The trajectory shown is conceptual. It represents one possible scaling relation between temporal integration (coherence) and representational capacity (model size), without implying a specific dynamical law. In practice, the exact growth dynamics depend on model design (eg, architectural constraints or learning paradigms such as frequentist or Bayesian updating, or the type of timewise compression) and functionality. The offset along the y-axis reflects the rather static capabilities associated with Level 1 systems. Temporal integration span (log T) sums over an index of discrete interactional turns (t), while allowing continuous time flow at basic levels of temporal integration. Model size R2 = (log T, log ∣Z∣).

In this context, it should be noted that agentic AI does not require full human or embodied experience. Rather, there appears to be a continuum, and a system’s capacity to engage patients—and thus its potential to elicit sustained involvement—will likely increase as a function of model capabilities (or of the lack of available treatment alternatives [10,11,40]). Conversely, this implies that systems with lower computational complexity (as described in Figure 2 and Tables 1 and 2), but with high levels of patient-facing autonomy, can be developed and may still exhibit a certain degree of clinical effectiveness [41]. Importantly, however, evidence on long-term effects remains limited [42], and many studies appear to be of very short duration, sometimes lasting only several weeks. Study quality also remains a concern [43].

**Table 1:** The 5-stage model of language-based artificial intelligence (AI) systems for psychiatry, psychotherapy, and counseling. Stages or abilities can overlap with categories of the taxonomy. The meaning of world model in the context of this table includes a combination of foundational model, world model, and agentic systems operating on the basis of them.

Level	Name	Definition	Core capabilities (examples)	Limitations
1	Knowledge level: static capability	Systems perform static benchmark tasks without real therapeutic interaction Comparable to written exams or synthesis of knowledge derived from the internet	Psychoeducation and exam knowledge Basic text analysis Predefined or simple dynamic responses	No adaptive behavior No therapeutic skills No time dimensionality
2	Elementary level: dynamic multi-turn consistency	Systems can execute individual evidence-based therapeutic microskills in dynamic dialogues Time: systems demonstrate timewise coherence within 1 dialogue or session Tactics: systems demonstrate tactical coherence over decisions within the dialogue World knowledge: systems possess some form of psychological world model and limited coherence in time	Conducting clinical interviews Applying clinical tests Multiturn consistency Within-session consistency Tactical decisions within dialogue Fluid application of standard techniques, such as cognitive restructuring, Socratic questioning, SMART^a^ goal setting, guided relaxation, or mindfulness and emotion-related techniques	Can manage 1 session No multisession coherence or adaptability No process monitoring No integrated macro perspective No case-level reasoning Can only be used as a specific tool within standard treatment Therapist oversees and structures treatment or tool application
3	Integration level: dynamic multisession consistency, for example, therapy assistant (blended therapy)	Systems can execute evidence-based therapeutic sequences over dynamic multisession contexts Time: systems exhibit multisession coherence (weeks) Operations: systems demonstrate operational coherence over decisions regarding therapy progress or dynamics World model: systems possess the ability to relate and update basic knowledge in person-specific psychological world models and demonstrate improved coherence in time. The system operates over important core elements of therapy	Within module: multisession treatment planning Basic between-module integration Basic information integration from clinical interviews and other sources Basic case conceptualization and macro perspective Basic person-specific/ideographic processing and memory Integration of early risk-flagging and basic procedural awareness Basic modality shifts Solid error and psychological bias profile over sessions	Certain gaps or shallow patterns are still evident Stops (relatively frequently) at ambiguity/risk Requires continuous human oversight or exhibits limited operability Not applicable for complex cases No or limited simulation or counterfactual capabilities
4	Saturation level: dynamic case-level consistency and advanced therapeutic system (therapist-in-the-loop or blended treatment)	Systems perform most therapeutic functions reliably over good parts of treatment Systems assess and communicate ambiguity to call for human supervision when needed Time: systems exhibit coherence over care path (months) Strategies: system demonstrates strategic coherence over complex therapy decisions World model: systems possess the ability to incorporate most person-, context-, and environment-related information required for typical cases or standard treatment	Case conceptualization and person-specific/ideographic modeling Dynamic adaptation of modules or interventions Ability to understand implicit or contradicting goals Integration of multiple modalities (eg, integrating diverse theoretical orientations) Simulation and counterfactual processing Advanced risk assessment and procedural awareness Solid error and psychological bias profile	Human supervision required occasionally (eg, crises, ethics, complexity, or handling of atypical cases [confirmation]) Cannot make high-stakes decisions Does not outperform humans Error rate requiring human support
5	Mastery: fully-capable agentic AI consistency over boundary conditions and rare cases	Systems can theoretically perform complete psychotherapy systems Systems can treat almost all cases, with near-optimal capabilities Time: systems exhibit coherence over the care path or even parts of biographies Strategies: systems demonstrate patient-specific strategic coherence over rare and extreme situations or cases World model: systems possess the ability to incorporate all person-, context-, and environment-related information required for all potential cases	System excels in therapy-related tasks Systems draw from large patient databases Deep relational modeling of patient-specific/ideographic information Advanced inference and adaptation to hidden states of patients and their environment (eg, counterfactual reasoning and social complexities) Systems demonstrate advanced implicit skills, such as agentic guidance capability Optimized bias and error profile Potentially full (private, medical, and social media) data integration (if desired)	Purely hypothetical with current technology Major ethical, legal, and regulatory complexities Major shifts across entire economic clusters are expected (not limited to mental treatment) Full clinical effectiveness of “digital only” treatment (on par with face-to-face treatment) is not impossible, but remains to be established^b^

^a^SMART: Specific, Measurable, Achievable, Relevant, and Time-Bound.

^b^Given the extensive literature on internet-based cognitive behavioral therapy, the default assumption would be that therapist-guided or blended formats will turn out to be more effective for the foreseeable future.

**Table 2:** Progression of artificial intelligence (AI)–based systems and potential future directions. Stages and functionalities may overlap between all 3 categories. While multiagent AI architectures already exist in several domains, their application in psychotherapeutic systems remains largely experimental at present. Level 4 and higher systems are conceptually probable, but exact designs will emerge according to future developments that can only be foreseen tentatively.

Dimension	Generative LLM^a^	AI agent (agentic AI)	Agentic MAS^b^	Agentic AI, MAS, or AI ecosystems
Description	Reactive dialogue system (eg, prompting)	Autonomous system that performs a specific task	Multiple coordinated or orchestrated AI agents for complex tasks and task integration	Highly advanced systems or ecosystems Multilayer agent ecosystem
Typical activities	Chatbot-based therapy (multiturn to multisession) Dynamic knowledge synthesis and presentation	Chatbot-based therapy (multisession and longer) Decision-system Planning-aid	Patient interface agent, external information integrator (eg, monitoring, sensory data), supervisor, strategist, risk manager, clinical knowledge provider, and orchestrator/moderator	Full integration of institutional guidelines Cross-case learning and pattern detection Mastery of rare cases Risk optimization (Derivation of new strategies)
Entity	Single component	Single agent architecture	Multiple internal agents	System-of-systems architecture
Dynamics and abilities	LLM-based dialogue system without or with limited externalized (short-term) memory or RAG^c^	Goal-directed adaptive behavior	Interactive, coordinated planning and reasoning, agentic group behavior, distributed decision-making, and learning	Optimized abilities and system-level adaptation, long-term learning and identity or relational tracking, neurosymbolic reasoning, inner representations and abstractions, and potential partial self-improvement (eg, self-play and pattern detection)
Time axis	Past-present	Present	Starting	Future
Agency level	Level 1	Level 2	Level 3	Level 4 and beyond

^a^LLM: large language model.

^b^MAS: multiagent systems.

^c^RAG: retrieval-augmented generation.

More specifically, to support effective treatment engagement—rather than merely increasing time spent with the program or the number of dialogues or sessions—agentic AI would need to integrate, optimize, and adapt treatment-relevant therapeutic mechanisms in a manner comparable to that of professional therapists. As a side note, although the evaluation of effective engagement would be comparatively easy to implement in AI therapy studies, we were unable to identify any studies that assessed it. Instead, most studies are short in duration, and the AI systems used clearly lack the computational complexity and temporal coherence required to approximate real therapist behavior.

Furthermore, this dissociation between high patient-facing autonomy and limited clinical effectiveness constitutes an important difference from the autonomous driving paradigm, even though both taxonomies follow a comparable 5-stage progression.

## Domain-Specific Benchmarking vs the Autonomous Driving Metaphor

Besides the technical implementation of required capabilities, one may also consider the properties of the domain to be mastered when assessing model requirements and the likely speed of technological development. The greater the computational demands, the more difficult it will be to achieve mastery in a given domain.

In this context, the Society of Automotive Engineers J3016 global standard is sometimes referenced in discussions of the future development of AI agents for therapy [44]. While this comparison may serve as a rough estimate of possible progression stages, several important differences should also be taken into account. Clear distinctions can therefore be drawn between the domains of autonomous driving and communication, or even therapy. With regard to computational bandwidth, processing speed, the type of information involved, and their dynamic interplay under bounded time constraints, as well as model requirements and the structure of the information itself (eg, number of model parameters, context length, number of tokens, AI architecture, model size, and temporal capacity), the 2 domains differ fundamentally.

More precisely, autonomous driving involves high bandwidth (eg, high visual resolution), high computational speed (as the vehicle must react within milliseconds), and predominantly geospatial information. This includes, but is not limited to, the real-time integration and prediction of potentially large numbers of traffic participants, random objects, weather and road conditions, traffic signs and rules, navigational decisions—including ethical dilemmas related to them—and the physical dynamics involved in steering a heavy object, to name only a few components (details are provided in Multimedia Appendix 1). A recent study by the Department of Electrical and Computer Engineering at the University of Houston [45] estimates that sensor data alone may amount to 1 terabyte per hour. Notably, this does not include data processing within external data centers.

By contrast, the psychological domain is structured very differently and therefore requires a different kind of computation. For example, language has comparatively low bandwidth, involving the serial processing of individual phonemes, and relatively low computational speed, with humans processing only a limited number of words per minute [46], amounting to far less than 1 gigabyte of information per hour of data extraction [47]. The challenge of dialogue, however, lies more in the semantic complexity of information, resulting in difficulties related to latent structure modeling, epistemic uncertainty, and coherence over time. More precisely, whereas geospatial navigation is a problem of real-time integration of large quantities of high-dimensional data, human dialogue is challenging because it requires the modeling of hidden structures and their interrelations. In this context, words, meanings, and intentions may be highly ambiguous and may therefore involve comparatively high informational uncertainty. A more detailed overview and comparison of both tasks—autonomous driving vs language-based dialogue—is provided in Multimedia Appendix 1. In summary, although hidden-state modeling is computationally demanding and remains an active area of development, autonomous driving still appears to be the more data-intensive domain [48].

For psychiatric practice, this implies that AI systems will likely be able to perform simpler tasks reliably. This consideration also helps explain why many white-collar occupations, despite their cognitive nature, appear at least as susceptible to AI-driven automation as traditional blue-collar jobs [49,50]. Uncertainty remains, however, particularly with regard to complex hidden-state modeling and coherence across the full course of therapy or across extended parts of a patient’s biography. In this regard, it may be useful to distinguish between full human experience and therapeutically effective information. While humans possess embodied experience shaped by the vividness of individual biographies and by highly complex phenomena such as consciousness or desire, digital therapeutics (such as internet-based interventions or iCBT) can be effective without, or with only minimal amounts of, these human qualities [37,38,51,52]. AI systems, therefore, do not necessarily require technical solutions to these remaining mysteries of modern science. Instead, they require dynamic human–machine interfaces capable of implementing therapeutic techniques or facilitating therapeutic change in a human-like manner. Importantly, this implies that effective AI agents do not require full human understanding or perspective-taking grounded in lived experience.

Constituting another important difference from autonomous driving, the psychological domain offers developers the “advantage” of currently unregulated off-label use, thereby increasing the volume of real-world data available for model training or fine-tuning. In addition, numerous adjacent domains relevant to psychology may also serve as sources for cross-domain learning.

Finally, a literal application of the Society of Automotive Engineers J3016 standard results in certain logical inconsistencies regarding the progression of autonomy levels, dynamic system requirements, user motivation, and end goals. The concept of autonomous driving is fundamentally oriented toward the user’s perspective. At level 3, the automated driving system (ADS) handles all driving tasks under specific conditions, but the human must remain ready to take over (human in the loop). At level 4, the ADS handles all driving tasks, and no driver intervention is required; the driver becomes a passive passenger. Psychological interventions differ fundamentally in this respect because patients are never merely passive passengers in the therapeutic process.

By contrast, successful therapy, by definition, requires patients to be highly engaged in the therapeutic process. This implies a major difference between the 2 logics: optimal AI agents in psychotherapy would not lead to passive users; rather, such systems should be optimized for the highest possible level of effective engagement. This includes techniques such as guided discovery, behavioral guidance, validation, confrontation, interpretation, empathic reflection, motivational interviewing, nudging, shaping, or emotional discovery.

Accordingly, terms such as AI-enabled guided discovery, AI-led behavioral guidance, or AI-supported motivational interviewing could be used to describe the phenomenon of agentic humans being guided by agentic AI. The overarching category for such abilities—in which agentic AI guides or fosters agentic human behavior with the aim of optimizing treatment engagement and outcomes—could be described using terms such as agentic guidance capability, dynamic change-facilitation, AI-facilitated engagement, or agentic change techniques. A list of potential expressions is provided in Multimedia Appendix 1. If agentic AI were to excel in such change-facilitation capabilities—through rich computational models and very long temporal coherence across treatments, years, or even longer—it could potentially unlock new therapeutic possibilities. In other words, the 2 fields clearly differ in their ultimate end goals.

A final difference worth noting is that level 4 ADS systems, such as the service offered by market leader Waymo, allow remote operators to intervene and take over control whenever the vehicle encounters ambiguous situations. From the user’s perspective, this remains consistent with the above definition, as the user continues to be a passive passenger. One might therefore argue that guided internet-based therapy already achieves a form of level 4 autonomy, insofar as it provides care with only minimal human support, including guidance on demand, while yielding outcomes comparable to face-to-face therapy [51,52]—even though most such systems currently remain rather static in design. In this regard, a recent meta-analysis did not identify clinically relevant differences between guidance-on-demand and regular-guidance formats [52]. However, it did find differences in patient motivation, further illustrating the distinction from the self-driving paradigm, in which passengers are passive consumers rather than human agents.

## Domain-Specific Benchmarking, Saturation, and Adaptation of Benchmarks

Benchmarking is currently the gold standard for evaluating the cognitive performance of AI systems within a given domain. In recent years, many general benchmarks have become saturated: their critical thresholds for success or failure have been surpassed, prompting developers to seek more challenging tasks capable of generating informative optimization signals. Popular examples of cognitive capabilities span a range of domains, including defeating world-class Go players, poker players, and professional gamers, as well as excelling in the Mathematical Olympiad or predicting protein structures with atomic precision.

To provide more detail on one recent example, practical mathematical and analytical tasks that were considered difficult only a few years ago are now often regarded as largely solvable. Leading mathematicians predict that AI will soon be used to generate machine-verified mathematical proofs (Terence Tao at the 2024 Mathematical Olympiad [53]). However, such systems still cannot invent entirely new mathematics. A persistent challenge lies in the real-time understanding and manipulation of logical analogies, as in few-shot learning, even though these tasks may at times appear trivial to many humans. For example, AI systems performed poorly on the so-called Abstraction and Reasoning Corpus (ARC) Challenge [54]. However, the ARC Challenge itself has also become a prominent example of recent benchmark saturation, which has led to the introduction of ARC-2 for fluid intelligence and ARC-3 for agentic tasks. For interested readers, the ARC Prize leaderboard provides a regularly updated overview of model performance [55].

But what exactly does benchmark saturation mean? Like many other performance measures (such as IQ tests), performance on AI benchmarks tends to follow an S-curve. This means that performance is initially low, then rises steeply, until further improvements occur at progressively lower rates, approaching an asymptote. When experts refer to saturated benchmarks, they do not usually mean that performance has mechanically reached 100% accuracy. Such a state would be inconsistent with the asymptotic performance patterns typically observed in stochastic AI systems and would suggest that the task has become effectively formalized.

Rather, saturation refers to performance levels approaching or exceeding human performance, or to a continued reduction in performance gains, indicating that most of the learning potential associated with a given task has already been realized. In other words, the task under evaluation no longer provides substantial opportunities for learning or optimization (at the chosen difficulty level), and developers must therefore seek new challenges for their models. For example, GPT-5 reached human-level performance on the medical subset of Massive Multitask Language Understanding [56], one of the most widely recognized benchmarks of common world knowledge currently available. Another example is the Graduate-Level Google-Proof Q&A Diamond benchmark, which comprises graduate-level questions in biology, physics, and chemistry. Notably, this benchmark was designed to minimize data contamination during training [57]. On this benchmark, Google’s Gemini Pro scored 92%, and ChatGPT 5.2 scored 86% in 2026 [58], whereas PhD-level experts achieve around 65% accuracy.

In this regard, a recent survey identified 283 representative benchmarks, spanning general, domain-specific, and target-specific evaluation settings, including clinically relevant domains. The survey also highlighted emerging efforts to address the saturation of static standard tests through more dynamic and multidimensional evaluation strategies [59]. For example, OpenAI introduced HealthBench in 2025 in partnership with 262 physicians, comprising 5000 realistic health conversations with multidimensional rubric-based evaluation and the explicit goal of maintaining unsaturated difficulty levels [60]. At the time of release, top-performing models still showed substantial room for improvement on this benchmark, underscoring the need for more ecologically valid and discriminative evaluation frameworks.

Taken together, there is clear evidence that model capabilities continue to increase steadily, and, as discussed above, human-level performance can often be reached on established (static) benchmarks. At the same time, substantial progress remains necessary with regard to dynamic testing, deep conceptual reasoning, ethical reasoning, and the integrated mastery of clinical knowledge. A particular challenge remains in the dynamic modeling of multiturn, session, or even multisession dialogues. Given the current dynamics of benchmark saturation, along with the fact that major AI developers are increasingly prioritizing higher-order reasoning capacities and more agentic forms of problem solving, any precise forecast remains difficult. Expert expectations continue to span a wide time horizon, ranging from several years to several decades, while past forecasts have often underestimated the pace of actual progress.

## Benchmarking AI Agents for Psychiatry, Psychotherapy, and Counseling

In the psychological and psychotherapeutic domain, the first dedicated benchmarks have recently been developed (Table 3) [59]. These can again be divided into static and multidialogue formats, although less dynamic benchmarks still predominate at present. In this context, the scope of this paper is limited to knowledge-based and dialogue-based systems for psychotherapy and counseling, rather than, for example, medical prescription or decision-support systems. Importantly, many of these approaches remain at a conceptual stage and either lack clinical results or report only preliminary findings. Furthermore, early multiagent frameworks have been introduced [61,62] to model therapists or to simulate clinical dialogues.

**Table 3:** Examples of recent benchmarks for psychotherapy, counseling, and psychiatry.

Benchmark	Year	Focus	Format
CounselBench [63]	2025	Counseling response quality and stress-testing with expert ratings	Single-turn counseling; includes 2000 expert evaluations
MentalBench-10	2025	Real-world mental health dialogue evaluation (Human vs multiple LLM^a^ responses)	10,000 conversations; 1 human + 9 LLM responses
CBT-Bench [64]	2025	CBT^b^ assistance fidelity (knowledge, application, and therapeutic process)	Benchmark suite for CBT-related tasks
MentalChat16K+ [65]	2025	Conversational mental health assistance plus structured counseling evaluation metrics	Dataset (16k+ QA^c^ pairs) plus 200-question evaluation benchmark
CPsyCoun [66]	2024	Multiturn psychological counseling reconstruction and automated evaluation	Multiturn dialogue reconstruction plus evaluation benchmark
MHQA^d^ [67]	2025	Mental health knowledge QA (multiple-choice and knowledge-intensive)	Multiple-choice dataset for benchmarking language models on mental health knowledge
TherapyGym [68]	2026	Evaluation and alignment of clinical fidelity in therapy interactions	Benchmark/evaluation setup
MindBench.ai [69]	2025	Transparent and standardized evaluation of mental health chatbots, including safety/harms	Open evaluation platform
C-SSRS^e^ Suicide Screening Evaluation [70]	2025	Suicide risk assessment/screening aligned with C-SSRS severity levels	Classification into a 7-level severity scale (0-6)
Large-scale mental health reliability benchmarks [71]	2025	Reliability and trustworthiness of LLMs in mental health contexts	Large-scale benchmark study
PsychEval	2026	Benchmark for psychological counseling	Multisession multitherapy benchmark

^a^LLM: large language model.

^b^CBT: cognitive behavioral therapy.

^c^QA: question answering.

^d^MHQA: Mental Health Question Answering.

^e^C-SSRS: Columbia–Suicide Severity Rating Scale.

In summary, benchmarks in this area are currently being developed at a rapid pace, and informative results can be expected in the near future. At present, however, benchmarking approaches show substantial heterogeneity in design, and more dynamic approaches would be desirable in order to assess practical capabilities more adequately. Given developments in general health AI, the emerging pattern of benchmark saturation and subsequent adaptation toward more demanding evaluation criteria appears likely, although much of this trajectory still lies ahead.

## A Taxonomy for Language-Based AI Agents

The taxonomy for language-based AI agents in psychotherapy, counseling, and psychiatry proposed here (Figure 2) reflects this empirical trajectory. It focuses on the technical capacity of systems to construct sufficiently accurate world models for a given task or developmental stage, based on the underlying foundation models involved (Tables 1 and 2). Although these stages are not entirely separable, they are rooted in empirical milestones that must be achieved on the path toward autonomous therapeutic agents.

Stage 1 (knowledge level) is defined by a model’s capability to provide information. Corresponding tests are designed to assess the quality of such knowledge provision. Technologically, LLMs are typically used to deliver this function. This level can already be considered largely mature for constrained tasks, as most systems today provide basic general knowledge reliably. In addition, in the above-mentioned domains of general cognitive performance, human experts already frequently lag behind AI systems in tasks involving knowledge provision. In psychotherapy, experiments have shown a preference for AI-generated responses over advice provided by mental health experts in blinded evaluations [72]. Taken together, current capabilities can be described as reflecting strong general benchmark performance, but not yet clinical mastery. The quality of clinical knowledge provision remains under investigation.

As a general indicator of performance on very difficult informational tasks, the success rate on the *Humanity’s Last Exam* increased from below 10% to 46% within the last year [73] (see the updated list of model performance on Wikipedia). The exam was designed in collaboration with leading scholars to include exceptionally difficult problems requiring very high levels of expertise across various scientific disciplines [74].

Stage 2 (elementary level, or single-technique level) assesses the capacity of LLMs or early agentic AI systems to solve dynamic tasks, such as conducting coherent multiturn dialogues [61,62,66,75]. Assessing specific therapeutic techniques—such as Socratic questioning [61], cognitive restructuring, or activity scheduling—is particularly well suited for this purpose. To perform these tasks, AI systems must possess at least some form of implicit world model of sufficient scope: dialogues must be realistic, and the generated content must be factually correct and contextually appropriate, grounded in reliable foundation models. For example, systems at this level demonstrate coherence over the course of a dialogue and can tactically decide what to focus on during the conversation. Technically, multiagent systems may also coordinate difficult tasks by implementing various subagents, such as an AI supervisor or AI orchestrator (Figure 1 and Table 1). However, Stage 2 models tend to have limited multisession coherence and only restricted patient-specific relational and contextual knowledge due to constraints related to data compression and context retention.

Thus, a system would qualify for level 2 if it were capable of conducting stable multiturn dialogue. In the cognitive domain, examples include Socratic questioning, cognitive restructuring, the downward-arrow technique, and work on core beliefs, rules, or assumptions. In the emotional domain, qualifying capabilities might include affect labeling, emotion rating, emotion detection, emotional awareness training, or the validation of emotions. In the behavioral domain, examples include activity planning, activity scheduling, relaxation training, and the management of homework assignments.

Stage 3 (integration level, or merging of domains) refers to multisession systems capable of integrating across various steps within a single domain (eg, introduction, application, repetition, microevaluation, and adaptation). Systems at this level may also demonstrate integration across domains or multiple pathways (eg, cognitive, emotional, or behavioral pathways), including capabilities such as problem solving or social support. At this stage, systems no longer apply isolated techniques in single sessions; instead, they can perform some degree of data integration, enabling basic patient-specific relational and contextual knowledge (patient-specific world models) as well as conceptualization at the level of therapy modules. To perform these tasks, systems require temporal coherence across multiple sessions. In addition, such models may demonstrate the ability to incorporate elements such as tests or diagnostic procedures into a simple macromodel.

Technically, advanced agents or multiagent systems can be implemented to coordinate different tasks across multiple sessions. This includes correct operations or dynamic adaptation over time. At the operational level, systems must choose between alternatives and plan across several steps. Foundation models underlying agentic AI may also begin to demonstrate initial sensitivity to irony or other implicit processes. Such models could start to generate accurate plan analyses, including the identification of simple conflicts, for example, within motivational schemes.

As a side remark, these capabilities can already be tested to a certain extent with current LLMs (eg, GPT-5), although plan analysis remains incomplete. In this regard, sycophancy—the model’s tendency to agree with the user in order to please them, rather than offering necessary challenge [76]—constitutes one current limitation. Furthermore, the plan analysis itself clearly requires further improvement.

Thus, systems would qualify for level 3 if they apply level 2 capabilities with greater depth and fluidity. They should be able to integrate, relate, and revisit content within and across techniques, and use information from one exercise during or in preparation for another. They should demonstrate the ability to relate techniques and integrate information in a temporally coherent manner within treatment modules or multiple sessions. This should enable more complex techniques, such as basic schema work, personalized self-compassion interventions, or confrontation, to name only a few examples. They should also be able to evaluate the success of applied modules or therapy segments both qualitatively and quantitatively, ideally with a focus on effective engagement. With regard to therapy planning and a broader macroperspective, level 3 systems may be capable of administering and evaluating questionnaires, clinical interviews, and anamnestic techniques in order to infer a basic therapy plan or identify treatment priorities. Such systems should also be able to indicate uncertainty and respond accordingly. Under certain conditions, with clear limitations and strong technical, legal, and supervisory safeguards, such systems could theoretically—or from a purely technical perspective—conduct some form of therapy (eg, blended therapy). Importantly, this is not a recommendation, but merely a technical description within the context of this taxonomy.

In Stage 4 (saturation level, or standard-case level), integration progresses to the point at which systems could, in principle and with only minor gaps, conduct most of therapy autonomously. Human therapists supervise the process and intervene only occasionally (“human in the loop”). In this context, several developmental trajectories appear plausible. As outlined in the previous sections, a distinction must be made between technical capabilities, therapeutic applicability, levels of clinical effectiveness, and effective engagement. This implies that certain Stage 4 systems could provide standard therapy with solid basic functionality. Such systems could be deployed in stand-alone or minimally guided formats, resulting in different levels of clinical effectiveness. Based on current research on guided and unguided internet-based therapy (eg, iCBT), one may assume that engagement and effectiveness would remain higher in guided and blended formats. Of course, Stage 4 stand-alone models would require very strong guardrails and extensive safety testing, as would any medical product.

At the same time, however, it seems plausible that powerful foundation models could enable even more advanced capacities, such as agentic guidance capability and other human-like therapeutic abilities. These may include the identification of unspoken schema conflicts, unrealistic expectations or plans, humor and irony, and guidance in complex social interactions or patterns of behavior that lie beyond the patient’s ability to verbalize them. At this stage, systems are expected to demonstrate not merely acceptable, but robust error and (psychological) bias-minimized performance, as well as the reliable indication of uncertainty, including the ability to call for human assistance when needed. Systems may also exhibit the capacity to detect and address alliance ruptures.

At the technical level, advanced multiagent systems—or even broader agentic ecosystems (systems of systems)—could be implemented to manage microprocesses, mesoprocesses, and macroprocesses in combination with powerful foundation models. Such systems should be able to coordinate therapeutic strategies over the entire course of treatment. They should be capable of selecting principal therapy strategies, planning interventions, implementing them, and adjusting them over the therapy horizon. As described in the following paragraphs, this implies a need for advanced data handling and compression, since computational requirements currently increase sharply as a function of model complexity and the number of operational steps (Figure 2, logarithmic scales). Such systems could implement advanced techniques, also including imagination (eg, conducting internal simulations) and reasoning in alternatives (eg, counterfactual reasoning or the representation of digital twins or typologies).

Thus, systems would qualify for level 4 if they exhibit strong interconnectivity across techniques and modules, as well as strong temporal coherence across the standard care pathway—from diagnostics to aftercare or booster sessions. In short, such systems would appear fully operational for the standard case. Capabilities could also enable flexible, nonlinear, or intermittent therapy plans, as well as advanced relapse management.

Stage 5 (mastery level) represents full technical capability. Systems at this stage can deliver therapy across a seamless care pathway from beginning to end, potentially including multiple courses of treatment. The advanced therapeutic techniques mentioned at Stage 4 could be further refined. For example, computationally powerful models could routinely run multiple simulations to optimize across virtual scenarios or counterfactuals, or draw on extensive databases or typologies. Systems at this level would exhibit technical therapeutic capabilities and rates of psychological bias or error rates that are comparable to (or potentially even better than) those of human clinicians. Human supervision could (theoretically) no longer be required, thereby enabling maximal scalability.

Importantly, however, this stage refers to technical implementation rather than necessarily to full clinical effectiveness. It is conceivable that even perfectly designed stand-alone systems may not achieve the same effectiveness as human-delivered psychotherapy, for example, because of the absence of lived experience. As argued in the introduction, such systems may remain tools rather than full substitutes for human therapists. Conversely, blended interventions may produce synergistic effects that purely autonomous systems cannot easily replicate. At the same time, one could also argue that full clinical effectiveness may eventually be achievable. This question, therefore, remains open and will need to be investigated in future research.

Importantly, such systems are currently hypothetical, and their development appears to require major technical innovations, for example, in handling computational complexity, data compression, neurosymbolic AI, self-optimization, and the identification of clear learning signals for gradient descent or recurrent learning.

This means that level 3 and level 4 types of assistants should be capable of delivering, or assisting in the delivery of, a substantial proportion of treatment-relevant structure and content, including but not limited to the following: dynamic cognitive restructuring and Socratic dialogue, schema-therapy-informed dialogues and exercises, just-in-topic interventions, tailored conflict analysis in social contexts, dynamic skills training of various kinds, the application of emotion-focused therapeutic techniques, dynamic training plans, as well as mindfulness- and ACT-informed interventions. Quantitative or qualitative symptom monitoring, together with corresponding hierarchical adaptation of relevant treatment tactics, operations, and strategies, is gradually implemented over the stages.

These capabilities can be understood as extensions of established therapeutic principles and may interact with common factors such as therapeutic alliance and patient engagement implemented in guided or blended formats. At the same time, therapists may guide treatment and benefit from the information provided by such systems, including in ways that improve their own clinical work. This constitutes another important difference between the domains of autonomous driving and psychological treatment. Importantly, these descriptions should be interpreted in the context of the technical challenges, implications for therapist training, and risks described below.

## Technical Challenges

Current technical challenges for psychological agentic AI include limited model size, temporal coherence, multimodal modeling, and the integration of hidden states, as well as difficulties in semantic, logical, epistemological, and relational analysis. These limitations result in reduced capacities for discourse modeling, personal world modeling, identity tracking, and logical consistency, as well as in semantic ambiguity and limited patient-specific relational and contextual models. In this context, personal knowledge graphs and social world models [77-79] can be used to represent and update relevant individual subjects or objects (via IDs), together with their attributes, relations, and corresponding timestamps, thereby contributing to patient-specific relational and contextual knowledge structures (eg, Kiara is the patient’s partner, they usually go running together on Wednesdays).

Several different approaches, ranging from more parallelized systems to more integrated architectures, have recently been proposed to achieve temporal integration or compression into stable person-specific models—for example, Larimar (episodic memory for LLMs [80]), HiMeS (Hippocampus-inspired Memory System [81]), and architectures developed for Character AI [82]. Another proposed concept for transforming the exponential data growth associated with the currently predominant transformer architecture into more linear growth is the recently introduced Extended Bidirectional Mamba architecture [83]. While such approaches could help address the central problem of data explosion and potentially lead to rapid advances, they currently remain largely conceptual and still exhibit limitations (eg, data accuracy at scale [83]).

In addition, there are studies that attempt to identify useful signals for model optimization or short-term feedback, for example, in therapist training [84-86]. Finally, personal world models do not depend on full physical modeling, but are instead rooted primarily in semantic and ideographic relational space, together with corresponding reasoning and integration capacities (eg, episodic, semantic, and emotional integration). However, physical or spatial modeling (eg, Genie 2 [87] or Li Fei-Fei’s World Labs) may still become relevant in the future for powerful foundation modeling, for example, for the stable identification or counting of objects in simulations.

Taken together, these technical challenges coexist with a growing number of proposed solutions. This makes any inference about the dynamics of future developments inherently difficult and contributes to diverging expert predictions. Patient-related uptake and acceptability, however, are likely to depend on perceived capabilities, temporal coherence, and thus on the overall attractiveness of these systems. Because many people already use LLMs for off-label psychological consultation, broad diffusion of this technology appears likely and may increase further with advances in personalization and memory capabilities.

## Implications for Standard Therapy and Education

Much could be said about the implications for the therapeutic process, as well as for the clinicians’ skills, education, and supervision required in AI-enabled workflows. For the purposes of this paper, however, we restrict ourselves to several brief considerations. First, therapist training is already undergoing adaptation or is beginning to prepare for these developments. For example, the first author (RS) of this paper is a member of the e–mental health task force of the Austrian Association for Cognitive Behavioral Therapists and is currently preparing instructor training workshops in this capacity. As technological advances continue, clinicians will increasingly need to supervise AI outputs, interpret uncertainty, and decide when to override system recommendations (see the Levels of Autonomy and Risk section).

Second, exact strategies for AI adaptation will vary depending on the institutional, regional, or national level of implementation of digital therapeutics and assistive tools. For example, some research clinics in Germany are already testing feature- or theory-based LLM evaluations of therapy sessions, with promising results [88,89]. At the same time, the understanding of implicit relational dynamics remains limited [90] for the reasons described above.

Third, the authors are currently considering the use of synthetic patients for teaching purposes at Graz University. Current internal evaluations suggest that such systems remain in a beta-developmental stage and are not yet fully ready for routine use. The systems tested may soon be capable of supporting basic dialogues, but they do not yet adequately cover special cases or difficult therapeutic situations, which may be of particular relevance for training.

## Levels of Autonomy and Risk

Applications in the clinical domain represent high-risk, high-stakes fields that require reliable functioning, error minimization, and high-quality risk management. AI cannot deliver care unless it is safe and embedded within an appropriate legal framework [91]. Examples of clinical risks include failures to detect suicidality or abuse, the amplification of dysfunctional cognitions through sycophancy [76], or failures to indicate ambiguity or uncertainty. In this regard, algorithmic explainability constitutes an important requirement, as therapists also need to understand how and why a given AI system arrived at a particular conclusion when performing a specific task [92]. In this context, it must be emphasized that minimizing errors or psychological biases is not equivalent to ethical justification, as current gold-standard treatments involve personal bonding, human understanding grounded in lived experience, and many other qualities that clearly remain within the human domain.

## Limitations

The following limitations should be considered when interpreting this paper. First, research on LLMs and agentic AI in mental health is an extraordinarily young and rapidly evolving field. This implies limited certainty regarding future directions and the pace of progress. Second, many recent citations (eg, on psychological benchmarks) have not yet been published in peer-reviewed journals but stem from public repositories. Their content should therefore be regarded as preliminary evidence. Third, although we attempted to differentiate scenarios of agentic AI progression at the intermediate stages of the taxonomy (eg, simpler programs for standard structured therapy vs powerful foundation models for a more engaging user experience), many other developmental pathways may emerge on the road toward AI-augmented treatment. Fourth, the stages and the capabilities described within them may overlap or, in some cases, be bypassed. Finally, for the sake of argument, we compared psychological interventions to white-collar occupations while largely setting aside more embodied therapeutic techniques.

It is also worth noting that initial taxonomy proposals already exist. For example, Stade et al [44] recently presented a 3-stage model explicitly inspired by levels of autonomous driving. While broadly convergent with our approach, their model is rooted more metaphorically in the general progression of autonomous driving than in empirical psychiatric benchmarking (Table 3 in Stade et al [44]). As argued in this paper, however, the 2 domains may differ substantially with respect to signal properties, required world models, temporal constraints, off-label use, and human–machine interaction at more advanced stages. The 5-stage model proposed here offers a more fine-grained resolution, providing more detailed information on developmental steps and AI capabilities. Another difference concerns the expected rate of development: Stade et al [44] assume a more linear trajectory with intermittent progress, whereas we maintain that, although fluctuations may occur at the micro level, the macro level potential for dynamic growth remains substantial.

Finally, it is important to emphasize that this framework is a taxonomy only, not a complete implementation model. The successful deployment of autonomous therapeutic systems will require additional structures and guidelines that extend far beyond technical capability. These include ethical standards, data-protection safeguards, regulatory and legal frameworks, as well as clear provisions for consumer information and consumer protection. Recent overviews suggest that adherence to holistic frameworks, such as the TEQUILA model addressing trust, evidence, and liability, will be essential for navigating these future challenges [93]. Moreover, robust governance mechanisms and interdisciplinary oversight will be necessary to ensure that such systems are introduced safely, transparently, and in alignment with public interests.

## Conclusion

In conclusion, we propose a 5-stage taxonomy applicable to language-based agentic AI in psychiatry, psychotherapy, and counseling. The taxonomy progresses from stage 1 (knowledge level), in which systems perform relatively static benchmark tasks, to stage 2 (elementary level), characterized by dynamic engagement in specific therapeutic microskills, and stage 3 (integration level), in which systems achieve case-level conceptualization. Stage 4 (saturation level) describes systems capable of partially autonomous functioning with varying degrees of complexity, whereas stage 5 (mastery level) represents AI systems that are technically capable of performing autonomous therapy, based on powerful foundation models and advanced semantic, relational, idiographic, and epistemological capacities.

We provide a fine-grained description of model capabilities for each stage of the taxonomy, covering the dimensions of computational complexity (including foundation models, world models, and agentic AI), together with progression in temporal coherence (from multiturn dialogue to multisession coherence to full care pathways), and, finally, the resulting therapeutic behavior (from tactical to operational to strategic behavior). In this context, we assign examples of typical capabilities to each stage of the taxonomy. Individual assignments may overlap or occur gradually, meaning that they are not always obligatory, but rather represent probable qualifiers of a given stage. The proposed taxonomy should therefore be understood as a general framework, with predictable deviations from its idealized progression. Accordingly, it seems plausible that autonomy may be achieved earlier in simpler systems with relatively narrow standard functionalities, provided that strong safety standards are in place.

At the same time, it appears desirable to develop systems with high computational complexity and advanced agentic capabilities that allow the approximation of more complex therapist behavior. In this context, we introduce the general concept of agentic guidance capability and provide examples such as AI-enabled guided discovery and AI-supported motivational interviewing. We further differentiate the end goal of autonomy by comparing it with the autonomous driving paradigm and by describing qualitative differences in what autonomy means in each domain (passive consumer vs active patient). On this basis, we conclude that the implementation of agentic guidance capability—and other high-level therapist skills—may be more relevant for effective engagement and outcome optimization than full autonomy itself, as such systems will likely be embedded within existing care structures and, for example, guided iCBT already constitutes an established, efficient, and scalable treatment pathway. Full autonomy, of course, offers maximal scalability and remains one of the long-pursued goals of digital therapeutics.

In this regard, high technical capability does not necessarily imply clinical effectiveness. Psychotherapeutic outcomes depend on multiple factors, including therapeutic alliance, expectancy, motivation, and other common factors, none of which can be reduced to correct task execution alone. Conceptually, AI systems may therefore be located within a 2D space, defined by their level of technical autonomy on one axis and their level of clinical effectiveness on the other. Progress along one dimension does not necessarily imply progress along the other.

## Appendix

Multimedia Appendix 1 Agentic AI capabilities index.

## Abbreviations

ADS: automated driving system
AI: artificial intelligence
ARC: Abstraction and Reasoning Corpus
HiMeS: Hippocampus-inspired Memory System
iCBT: internet-based cognitive behavioral therapy
ICMAS: International Conference on Multi-Agent Systems
JITAI: just-in-time adaptive intervention
LLM: large language model
WHO: World Health Organization
