# Supplementary material for: AI Agents Are Coming: 5-Stage Taxonomy of Language-Based AI Systems for Psychiatry, Psychotherapy, and Counseling
Source: JMIR Ment Health. 2026 Jul 13;13:e91746. doi: 10.2196/91746 (PMC13408468; doi:10.2196/91746)
Supplement: Multimedia Appendix 1 [file mental_v13i1e91746_app1.docx]

1. **Selected list:**

- **agentic guidance capability**
- **dynamic change-facilitation**
- **AI-facilitated engagement**
- **agentic change techniques**
- **AI-enabled guided discovery**
- **AI-led behavioral guidance**
- **AI-supported motivational interviewing**

**Full list:**

1. **1. Capability-level terms**

- change-facilitation capability
- engagement-facilitation capability
- relational guidance capability
- supportive guidance capability
- therapeutic guidance capability
- therapeutic interaction capability
- **agentic guidance capability**
- **dynamic change-facilitation**
- **AI-facilitated engagement**
- **agentic change techniques**
- **effective engagement**
- **human-like therapeutic abilities**
- **technical therapeutic capabilities**
- **therapeutic capabilities**
- **treatment-relevant therapeutic mechanisms**

1. **2. Process / outcome terms**

- AI-assisted behavior change
- AI-enabled change processes
- AI-enabled engagement processes
- AI-enabled human change
- AI-enabled human change processes
- **AI-enabled guided discovery**
- AI-facilitated adaptive change
- AI-facilitated human change
- AI-facilitated patient agency
- AI-facilitated patient engagement
- AI-facilitated self-directed change
- AI-facilitated therapeutic change
- AI-facilitated therapeutic engagement
- AI-facilitated transformation
- AI-fostered engagement strategies
- AI-guided agentic engagement
- AI-guided behavior change
- AI-guided change
- AI-guided change processes
- AI-guided human change
- AI-guided motivation
- AI-guided motivational techniques
- AI-guided patient engagement
- AI-guided therapeutic engagement
- **AI-led behavioral guidance**
- AI-mediated human change
- AI-supported behavior change
- AI-supported change processes
- AI-supported effective engagement
- AI-supported human change
- AI-supported motivational interviewing
- AI-supported patient engagement
- AI-supported self-agency
- AI-supported self-directed change
- AI-supported self-efficacy
- AI-supported self-effectiveness
- AI-supported therapeutic change
- **guided discovery**
- **behavioral guidance**
- **validation**
- **confrontation**
- **interpretation**
- **empathic reflection**
- **motivational interviewing**
- **nudging**
- **shaping**
- **emotional discovery**
- **Socratic questioning**
- **cognitive restructuring**
- **downward-arrow technique**
- **work on core beliefs**
- **work on rules or assumptions**
- **affect labeling**
- **emotion rating**
- **emotion detection**
- **emotional awareness training**
- **validation of emotions**
- **activity planning**
- **activity scheduling**
- **relaxation training**
- **management of homework assignments**
- **problem solving**
- **social support**
- **basic schema work**
- **personalized self-compassion interventions**
- **dynamic adaptation of modules or interventions**
- **simulation and counterfactual processing**
- **advanced risk assessment**
- **procedural awareness**
- **guidance in complex social interactions**
- **ability to understand implicit or contradicting goals**
- **quantitative symptom monitoring**
- **qualitative symptom monitoring**
- **hierarchical adaptation of treatment tactics, operations, and strategies**

1. **3. Relational agentic AI**

- agentic co-regulation capability
- agentic guidance dynamic
- agentic relational capability
- agentic therapeutic rupture repair
- relational agency capability
- relational change capability
- **deep relational modeling**
- **person-specific ideographic modeling**
- **person-specific psychological world models**
- **patient-specific relational and contextual knowledge**
- **therapeutic alliance**
- **alliance rupture detection**
- **addressing alliance ruptures**
- **identity tracking**
- **personal world modeling**
- **social world models**
- **personal knowledge graphs**

1. **4. Agent-to-agent capability**

- agent-to-agent change facilitation
- agent-to-agent engagement
- agent-to-agent facilitation
- agent-to-agent guidance
- agent-to-agent influence
- agent-to-agent interaction
- agent-to-agent interaction capability
- agent-to-agent support
- agent-to-agent therapeutic influence
- AI-enabled human guidance capability
- AI-mediated agentic interaction
- AI-to-human interaction capability
- human-directed agentic capability
- human-directed agentic support
- inter-agent capability
- inter-agent guidance capability
- **AI supervisor**
- **AI orchestrator**
- **patient interface agent**
- **external information integrator**
- **supervisor**
- **strategist**
- **risk manager**
- **clinical knowledge provider**
- **orchestrator / moderator**
- **multiple coordinated or orchestrated AI agents**
- **distributed decision-making**
- **agentic group behavior**
- **agentic ecosystems**
- **multi-layer agent ecosystem**
- **system-of-systems architecture**
- **multiple internal agents**

1. **5. Agentic AI-to-human change**

- agentic AI–facilitated engagement
- agentic AI–human change facilitation
- agentic AI–supported behavior change
- agentic AI-mediated behavior change
- **AI-facilitated engagement**
- **effective engagement**
- **dynamic adaptation of modules or interventions**
- **simulation and counterfactual processing**
- **advanced inference and adaption to hidden states**
- **guidance in complex social interactions**
- **ability to understand implicit or contradicting goals**
- **patient-specific strategic coherence**
- **strategic coherence**
- **operational coherence**
- **tactical coherence**
- **temporal coherence**
- **multi-session coherence**
- **dynamic case-level consistency**
- **dynamic multi-session consistency**
- **case-level conceptualization**

1. **6. Capability level**

- therapeutic guidance capability
- change-facilitation capability
- engagement-facilitation capability
- **agentic guidance capability**
- **dynamic change-facilitation**
- **AI-facilitated engagement**
- **agentic change techniques**

1. **7. Process level**

- AI-facilitated therapeutic engagement
- AI-supported human change
- AI-guided behavior change
- AI-supported therapeutic change
- AI-facilitated human change
- **AI-enabled guided discovery**
- **AI-led behavioral guidance**
- **AI-supported motivational interviewing**
- **effective engagement**
- **case-level conceptualization**

**Extended list:**

1. **1. General CBT / process-guiding terms**

- AI-enabled guided discovery
- AI-supported guided discovery
- AI-guided Socratic questioning
- AI-facilitated cognitive restructuring
- AI-guided cognitive restructuring
- AI-supported cognitive conceptualization
- AI-guided action planning
- AI-supported action planning
- AI-facilitated homework planning
- AI-guided homework planning
- AI-supported progress monitoring
- AI-guided progress monitoring
- AI-led behavioral guidance
- AI-facilitated behavioral guidance
- AI-supported structured therapeutic guidance
- AI-guided change-strategy guidance

1. **2. DBT-related expressions**

- AI-supported skills coaching
- AI-guided skills coaching
- AI-facilitated skills generalization
- AI-guided skills generalization
- AI-supported chain analysis
- AI-guided chain analysis
- AI-facilitated solution analysis
- AI-guided solution analysis
- AI-supported contingency management
- AI-guided contingency management
- AI-facilitated commitment strategies
- AI-guided commitment strategies
- AI-supported validation-based guidance
- AI-guided dialectical guidance
- AI-facilitated consultation to the patient
- AI-supported crisis coaching
- AI-guided crisis coaching
- AI-facilitated behavioral rehearsal
- AI-guided therapist-like skills application

1. **3. Schema-therapy-related expressions**

- AI-supported limited reparenting
- AI-guided limited reparenting
- AI-facilitated empathic confrontation
- AI-guided empathic confrontation
- AI-supported mode work
- AI-guided mode work
- AI-facilitated mode-guided intervention
- AI-guided mode-guided intervention
- AI-supported mode dialogues
- AI-guided chair work
- AI-facilitated chair dialogues
- AI-supported imagery rescripting
- AI-guided imagery rescripting
- AI-supported limit setting
- AI-guided limit setting
- AI-facilitated therapy relationship work
- AI-supported corrective emotional experience
- AI-guided Detached Protector confrontation
- AI-guided Punitive Parent confrontation
- AI-guided Demanding Parent confrontation
- AI-supported schema-focused guidance

1. **4. ACT / contextual CBT expressions**

- AI-supported values clarification
- AI-guided values clarification
- AI-facilitated committed action coaching
- AI-guided committed action
- AI-supported acceptance coaching
- AI-guided acceptance coaching
- AI-facilitated defusion coaching
- AI-guided defusion coaching
- AI-supported choice-point guidance
- AI-guided choice-point work
- AI-facilitated present-moment guidance
- AI-guided present-moment guidance
- AI-supported psychological flexibility coaching
- AI-facilitated values-based behavior change
- AI-guided action-plan guidance
- AI-supported recommitment guidance

1. **5. Personality-disorder / high-structure treatment expressions**

- AI-supported emotion regulation coaching
- AI-guided emotion regulation coaching
- AI-supported interpersonal effectiveness coaching
- AI-guided interpersonal effectiveness coaching
- AI-facilitated impulse-control coaching
- AI-guided impulse-control coaching
- AI-supported rupture-repair guidance
- AI-guided rupture repair
- AI-facilitated pattern interruption
- AI-guided behavioral pattern breaking
- AI-supported needs-oriented limit setting
- AI-guided corrective mode coaching
- AI-supported self-soothing guidance
- AI-guided high-conflict interaction guidance

1. **Capability-style wording**

- AI-enabled skills-coaching capability
- AI-enabled chain-analysis capability
- AI-supported contingency-management capability
- AI-guided mode-work capability
- AI-facilitated imagery-rescripting capability
- AI-enabled values-clarification capability
- AI-supported emotion-regulation coaching capability
- AI-guided interpersonal-effectiveness capability
- AI-enabled rupture-repair capability
- AI-supported limit-setting capability

1. **Agentic wording**

- agentic AI-supported skills coaching
- agentic AI-guided chain analysis
- agentic AI-facilitated empathic confrontation
- agentic AI-supported imagery rescripting
- agentic AI-guided values clarification
- agentic AI-supported committed action
- agentic AI-guided emotion regulation coaching
- agentic AI-supported interpersonal effectiveness coaching
- agentic AI-facilitated rupture repair
- agentic AI-guided corrective mode coaching

**Extended list selections**

- AI-supported skills coaching
- AI-guided chain analysis
- AI-supported contingency management
- AI-facilitated empathic confrontation
- AI-supported limited reparenting
- AI-guided mode work
- AI-supported imagery rescripting
- AI-guided limit setting
- AI-supported values clarification
- AI-facilitated committed action coaching
- AI-guided emotion regulation coaching
- AI-supported interpersonal effectiveness coaching
- • AI-facilitated rupture repair
